# Investigation of the Partial Permittivity of Rigid Polyurethane Foams by a Circular One-Side-Access Capacitive Sensor

**DOI:** 10.3390/polym17050602

**Published:** 2025-02-24

**Authors:** Ilze Beverte

**Affiliations:** Institute for Mechanics of Materials, University of Latvia, LV-1004 Riga, Latvia; ilze.beverte@lu.lv; Tel.: +371-29-46-42-50

**Keywords:** polyurethane foams, capacitive sensor, one-side access, partial permittivity, model functions, rate of change, surface charge

## Abstract

The determination of the surface charge density distribution and the transcapacitance of capacitive one-side-access circular sensors with three electrodes on the active surface remains problematic both theoretically and experimentally. To provide an input, a novel experimental study was carried out on the partial permittivity of rigid PU foams by means of a capacitive circular OSA sensor with three electrodes on the active surface. An original and effective method was elaborated in order to determine the model functions of the obtained experimental data of the partial permittivity. A numerical estimation for the rate of change in the partial permittivity was made and the highest rate of change was determined. It was identified that the highest rate of change takes place at the inter-electrode zone and depends on the density and the true permittivity in a nonlinear mode, approximated with second-order polynomials. The overall character of the rate of change in the partial permittivity in the dependence of the radius of the covered area was found to be comparable to that of the surface charge density distribution curve, estimated theoretically for a circular two-electrode OSA sensor. The experimental results on the partial permittivity can be useful in the performance evaluation and design of the optimal proportions of capacitive circular OSA sensors, as well as in the verification of the corresponding mathematical models.

## 1. Introduction

Rigid PU foams are a “polymer–gas” composite, which has the same polyurethane matrix at any density of foams, 30 kg/m^3^ ≤ ρ ≤ 1350 kg/m^3^ [1,2,3], which ensures a similar mechanism of polarization and monotonously increasing permittivity at an increasing density. Polyurethanes make up a group of generally polar polymers with a surface free energy ≈ 40 mJ/m^2^ [2,4]. For PU foams of low density (30 kg/m^3^), the permittivity is 1.065; for PU foams of medium density (550 kg/m^3^), the permittivity is 2.10, and, for monolithic polyurethane (1280 kg/m^3^), the permittivity is 3.35 (1 kHz). The foams have low dielectric interference and nearly non-dispersive permittivity [4]; thus, a good dielectric performance can be ensured in a wide frequency range. That makes rigid PU foams an appropriate material for aerospace and automotive components, electronics packaging, electric insulators, and radomes (protective structures for outdoor antennae, locators, and telescopes), providing a radio-frequency transparent layer along dimensional stability.

In the non-destructive evaluation of dielectric materials, in the frequency band up to 10 MHz, the capacitance method is one of the main testing methods. Capacitive one-side-access (OSA) sensors [5,6,7] are used in the non-destructive testing and characterization of dielectric materials and products. The sensors have a potential to detect the quality and contamination of food, rebars in concrete, corrosion under insulation, water intrusion into composite structures, cracks, delamination, impact damage, etc. [8,9,10,11]. Several problems exist when working with capacitive OSA sensors: they excite test objects with a non-homogeneous electric field and have low sensing capacitances and high stray capacitances [12]. Contrary to the parallel-plate capacitive sensors [5,6], working with a homogeneous electric field, there are no simple formulae for calculating the sensing capacities of OSA sensors [5,12,13].

Numerical results of several mathematical models, based on the Laplace equation for the electrostatic potential and boundary conditions, are available for circular OSA sensors with two electrodes [14,15,16,17,18]. The calculated curves for the surface charge density with a dependence on the radial dimension of the active area are depicted in [14,18]. A simplification is assumed in [17] regarding an even distribution of the surface charge on the sensor’s electrodes, a disk, and an annulus. In [19,20], a mathematical model for a circular three-electrode OSA sensor is elaborated, but the numerical results for the surface charge distribution are lacking.

In [14], the sensor’s surface charge density distribution was calculated utilizing the spatial domain Green’s functions and the method of moments. It is outlined that the regions of an OSA sensor which contribute most to the sensor’s capacitance are the outer edge of the inner electrode and the inner edge of the outer electrode, where the surface charge density is the highest [5,14,18].

A shortage of experimental data related to the surface charge density distribution over the active surface of the capacitive circular OSA sensors was identified. The experimental studies on the properties of dielectrics are carried out on complete samples, i.e., such samples which fill the working volume of a circular OSA sensor completely [4,5,14,21,22]. A measurement of the permittivity on a complete sample provides a single value, which characterizes the overall effect of the surface charge on the dielectric sample and is a material constant [4,5,6,7,22,23]. It is of interest what information can be gained from technically the same measurements with an OSA sensor at partial samples, i.e., samples, which cover the active area of an OSA sensor partially, for less than 100%. Empirical knowledge on how the results of the measurements depend on the dimensions of the partial samples would facilitate the performance evaluation, design, and modelling of the optimal proportions of OSA capacitive sensors [23,24,25,26,27,28,29].

An analytical expression is known for the capacitance of a parallel-plate capacitor, partly filled with a dielectric. A dielectric parallelepiped of a size equal to the size of the working volume of the capacitor is slid gradually in between the plates at constant increments Δx along the transversal dimension ox of the capacitor. Treating the system as several capacitors, connected in parallel, the equivalent capacitance is determined for each x [30]. The increments ΔC of the equivalent capacitance, corresponding to each increment Δx = const., are constant, suggesting an even distribution of the surface charge density.

In [31], the concept of constant increments was adapted for capacitive circular OSA sensors as equal increments of coverage of the active area. Partial electric susceptibility (further-partial susceptibility) was investigated experimentally at a partial coverage of a circular OSA sensor with rigid PU foams. The partial samples were designed to repeat the circular symmetry of the OSA sensor’s active area. The implementation of the relative partial susceptibility permitted the transformation of susceptibility data to a common scale of 0.0–1.0 and to outline the main trends. The overall character of the rate of change in the relative partial susceptibility was found to be similar to the character of the surface charge density distribution curves, obtained from mathematical modelling [5,14].

The study is aimed at an investigation of the partial permittivity of rigid polyurethane foams and monolithic polyurethanes. Model functions of the experimental data of (1) the partial permittivity and (2) the relative partial permittivity as well as of their rate of change are determined, based on the model functions for the foams’ relative susceptibility. The rate of change in the partial permittivity with a dependence on the radius of the cylindrical subsamples is estimated and compared to the surface charge density distribution, resulting from mathematical modelling. It is shown that the highest rate of change depends on the density and the true permittivity of the PU foams in a nonlinear mode, approximated with second-order polynomials.

## 2. Materials and Methods

Two groups of dielectric materials are considered: group 1 comprising low- to medium-density PU foams SikaBlockM80, SikaBlockM150, and SikaBlockM450 with similar true permittivity values 1.00 < ε < 1.80 and group 2 with low- to medium-density PU foams SikaBlockM80 and SikaBlockM450, lab-made PU, industrial PU SikaM945, and a comparative material, epoxy Lab975 New, with significantly differing true permittivity values 1.0 < ε < 9.0, Table 1 (SikaBlockM80 and SikaBlockM150 are included in both groups for a better comparison). Rigid PU foams are a cellular composite of nearly 98% of closed-cells [1,2,3]. It is assumed that the gaseous phase is the same for all densities of the foams. Similar to the PU foams, the epoxy Lab975 New is a polar dielectric. Except for the lab-made PU [4], all dielectric materials were acquired at Sika JSC (Baar, Switzerland).

A sample is considered to be complete if its (1) cross-sectional surface covers the entire active area of the sensor and (2) thickness equals to or exceeds the penetration depth of the electric field of a certain frequency into the given dielectric. The complete samples have to be thick enough to provide the true permittivity; therefore, 3–4 times the thickness of penetration depth was used as appropriate thickness for PUR foams’ samples, t ≈ 20–25 mm [21]. The subsamples were made from complete samples by consecutively removing shells of certain thickness of walls [31].

Circular cylinders and shells (complete samples and subsamples) were made to study permittivity with a dependence on the coverage of the active area of OSA capacitive sensor, characterized by the coverage coefficients k_c_ and k_s_:

(1) $$k_{c}=\frac{S_{c}}{S_{0}}={\left(\frac{d_{c}}{D_{0}}\right)}^{2}={\left(\frac{r_{c}}{R_{0}}\right)}^{2},\;k_{s}=\frac{S_{s}}{S_{0}}=\frac{\pi \left(D_{0}^{2}-d_{sin}^{2}\right)}{\pi D_{0}^{2}}=1.0-{\left(\frac{d_{sin}}{D_{0}}\right)}^{2}=1.0-{\left(\frac{r_{sin}}{R_{0}}\right)}^{2},d_{sin}=d_{sout}-t,\;0.0\leq r_{c},r_{sin}\leq R_{0}\;and\;0.0\leq k_{c},k_{s}\leq 1.0,$$

where D_0_ = 45 mm—diameter of a complete cylindrical sample, d_c_—diameter of a cylindrical subsample (d_c_ ≤ D_0_), r_c_ = d_c_/2—radius of a cylindrical subsample (r_c_ ≤ R_0_), d_sin_ and d_sout_ = D_0_—the inner and the outer diameter of a shell (d_sin_ ≤ d_sout_), r_sin_ = d_sin_/2—the inner radius of a shell (r_sin_ ≤ R_0_), t—thickness of the shell wall, S_c_—cross-sectional area of a cylindrical subsample, S_s_—cross-sectional area of a shell (the walls), and the total active area of the sensor S_0_ = πD_0_^2^/4 = 1590.4 mm^2^ [31]. The samples and subsamples were made manually with a jewellery arc-saw, and then processed with a sand paper. The coverage of the active area of a circular OSA sensor (1) equals 100% for the complete samples and (2) is less than 100% for the subsamples. The linear dimensions were measured with a calibrated calliper Mitutoyo Vernier A3 (Mitutoyo Europe GmbH, Neuss, Germany), with range 0–200 mm, resolution 0.01 mm, and accuracy 0.012 mm.

**Figure 1:**
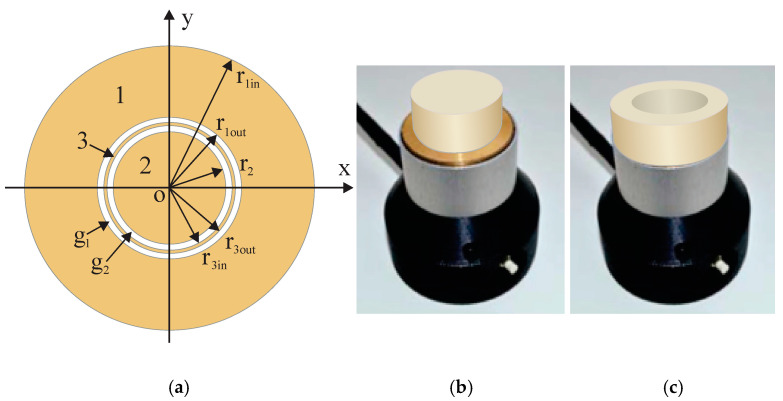
(**a**) A general scheme of the active area of circular OSA sensor [12] with the electrodes: driven 1, sensing 2, and guard 3; g_1_ and g_2_—gaps 1 and 2. The inner and outer radii r_1in_ = 10.5 mm and r_1out_ = 22.5 mm of the electrode 1, radius r_2_ = 8.4 mm of the electrode 2, and the inner and outer radii r_3in_ = 8.95 mm and r_3out_ = 9.95 mm of the electrode 3. PU foams’ subsamples on the active area: (**b**) a cylinder and (**c**) a shell. PU foams’ subsamples on the active area: (**b**) a cylinder and (**c**) a shell.

Measurements were made [4,21,22,31] with an experimental dielectric spectrometer equipped with a capacitive sensor of OSA type [12]. A dielectric sample, complete or partial, was placed on the active area of the sensor (Figure 1a–c) and excited via electrodes, with electric field generated by sinusoidal voltage signals [6,7,32]. The sensing electrode, the reference capacitor, and the unity gain buffer amplifier were covered with a screen in order to carry out stray-immune capacitance measurements. The screen forms a guard electrode on the active area around the sensing electrode, as shown in Figure 1a. The guard electrode is fed by the same voltage as the sensing electrode (the active guarding) through a voltage follower, thus suppressing the electric field between the driven electrode and the sensing electrode outside the sensor’s active surface. The diameter of the active area D_0_ = 45.0 mm and radius R_0_ = 22.5 mm; amplitude value of the sinusoidal excitation signals U_0_ = 20 V. The signals were generated at discrete frequencies, increasing in a geometric progression:

f_n_ = f_1_, 2f_1_, …, 2(n − 1)f_1_ Hz, where f_1_ = 10 Hz, n = 1, 2, …, 16, and f = 10, 20, …, 327, 680 Hz. (2)

It was shown that, for PU foams and monolithic polyurethanes, the loss part ε^″^(f) of the complex permittivity $\tilde{\varepsilon }(jf)=\varepsilon^{\prime }(f)-j\varepsilon^{\prime \prime }(f)$ is small compared to the real one $\varepsilon^{\prime }(f)$ [21]. Then, $\tilde{\varepsilon }(jf)\approx \varepsilon^{\prime }(f)$ and the real part $\varepsilon^{\prime }(f)=\varepsilon (f)$ is referred to as permittivity. Since the dielectric dispersion of PU foams and monolithic polyurethanes is small in the given frequency range [4], permittivity is analyzed further at one frequency f = 1/2($f_{7}$ + f_8_) = 960 Hz ≈ 1 kHz.

The true permittivity values, measured with the OSA sensor and the spectrometer, were compared to those measured with a Broadband Dielectric Spectrometer BDS-50 (Novocontrol Technologies GmbH & Co. KG, Montabaur, Germany), comprising a parallel-plate capacitor. Rigid PU foams (densities 95, 152, and 222 kg/m^3^) and monolithic polyurethane (density 1280 kg/m^3^) were tested. The relative difference between values provided by the two apparatuses was less than ≈5% which proved high accuracy of the OSA sensor and the spectrometer [4].

The spectrometer and test materials were situated in the same premises for entire study to attain thermodynamic equilibrium. The temperature regime in the test premises was T = 21 °C ± 2 °C, and that of the relative humidity RH = 45% ± 10%. No conditioning was made for samples. Accuracy of the spectrometer in permittivity measurements, in conditions of repeatability, was estimated with the expanded uncertainty U_S_95.45% = ±0.01 [4,21]. Three consecutive measurements of permittivity spectra were made for each data point. Calibration of the spectrometer was made before each measurement, with respect to permittivity value, delivered by the sensor in air. Permittivity spectra were approximated according to methodology [21]. More information on several concepts of the study is available in [31].

The reading of spectrometer for (a) complete samples is denoted further as the true permittivity ε_t_ = ε (the material constant) and for (b) subsamples as the partial permittivity ε_p_ (the term “the measured permittivity” is used for the partial permittivity in [31]). Susceptibility, calculated for the complete samples, is denoted as the true susceptibility χ_t_ and the value, calculated for the subsamples as the partial susceptibility χ_p_. For the light- to medium-density PU foams of group 1, the true permittivity and the partial permittivity were determined for both cylinders and shells. For dielectrics of group 2, permittivity values were determined only for the cylindrical subsamples, due to technical challenges when making the shells.

## 3. Theoretical

### 3.1. The Relative Partial Permittivity

A relation is known, ε = 1.0 + χ [33], or, in the present notations, ε_t_ = 1.0 + χ_t_. Then, the partial permittivity ε_pc_ and ε_ps_ for cylinders and shells is linked to the partial susceptibility χ_pc_ and χ_ps_ for cylinders and shells as follows:

ε_pc_ = 1.0 + χ_pc_ and ε_ps_ = 1.0 + χ_ps_. (3)

The relative partial permittivity RE is determined by normalizing ε_pc_ and ε_ps_ with ε_t_:

(4) $${RE}_{c}\left(r_{c}\right)=\frac{\varepsilon_{pc}\left(r_{c}\right)}{\varepsilon_{t}}=\frac{1.0+\chi_{pc}\left(r_{c}\right)}{\varepsilon_{t}}\;and\;{RE}_{s}\left(r_{sin}\right)=\frac{\varepsilon_{ps}\left(r_{sin}\right)}{\varepsilon_{t}}=\frac{{1.0+\chi }_{ps}\left(r_{sin}\right)}{\varepsilon_{t}},$$

where r_c_ is the radius of a dielectric cylinder and r_sin_ is the inner radius of a dielectric shell, as seen in Figure 1b,c. RE_c_ and RE_s_ characterize the fraction, for which the partial permittivity of a cylinder or a shell forms from the true permittivity of a complete sample. The relative partial susceptibility RΧ was defined in [31] by normalizing the partial susceptibility χ_p_(r) for cylinders and shells with χ_t_:

(5) $${RX}_{c}\left(r_{c}\right)=\frac{\chi_{pc}\left(r_{c}\right)}{\chi_{t}}\;and\;{RX}_{s}\left(r_{sin}\right)=\frac{\chi_{ps}\left(r_{sin}\right)}{\chi_{t}}.$$

Let us divide the numerator and denominator of Equation (4) by the true susceptibility χ_t_:

(6) $${RE}_{c}\left(r_{c}\right)=\left(\frac{1}{\chi_{t}}+\frac{\chi_{pc}}{\chi_{t}}\right)\frac{\chi_{t}}{\varepsilon_{t}}=\frac{1}{\varepsilon_{t}}+\frac{\chi_{t}}{\varepsilon_{t}}RX\left(r_{c}\right)=\frac{1}{\varepsilon_{t}}\left[1+\chi_{t}RX(r_{c})\right]\;and\;{RE}_{s}\left(r_{sin}\right)=\left(\frac{1}{\chi_{t}}+\frac{\chi_{psin}}{\chi_{t}}\right)\frac{\chi_{t}}{\varepsilon_{t}}=\frac{1}{\varepsilon_{t}}+\frac{\chi_{t}}{\varepsilon_{t}}RX\left(r_{sin}\right)=\frac{1}{\varepsilon_{t}}\left[1+\chi_{t}RX(r_{sin})\right].$$

Then, the relative partial susceptibility is expressed via the relative partial permittivity as follows:

(7) $${RX}_{c}\left(r_{c}\right)=\frac{1}{\chi_{t}}\left[{\varepsilon_{t}RE}_{c}\left(r_{c}\right)-1\right]\;and\;{RX}_{s}\left(r_{sin}\right)=\frac{1}{\chi_{t}}\left[{\varepsilon_{t}RE}_{s}\left(r_{sin}\right)-1\right].$$

When r_c_ = 0.0 mm or r_sin_ = R_0_, there is no sample on the active area. The sensor is in the air: ε_t_ = ε_air_ = 1.0006 and RX ≈ 0.0. Then, RE_c_ = RE_s_ ≈ $\frac{1}{\varepsilon_{t}}$ and, consequently, 1/ε_t_ ≤ RE_c_ and RE_s_ ≤ 1.0. The dependence of RX_c_ on RE_c_, when RE_c_ increases from 0.0% to 100%, was calculated numerically for the dielectric materials, listed in the Table 1. The graphs “RX_c_-RE_c_” were constructed and compared.

### 3.2. Model Functions

To smooth the local deviations and outline the main trends for the experimentally acquired relationships “ε_cp_(r_c_)-r_c_” and “ε_ps_(r_sin_)-r_sin_”, let us implement model functions Φ1(r_c_) and Ψ1(r_sin_):

Φ1(r_c_) ≈ ε_pc_(r_c_) and Ψ1(r_sin_) ≈ ε_ps_(r_sin_). (8)

To avoid the complex and time-consuming direct determination of the mode and parameters of Φ1(r_c_) and Ψ1(r_sin_), it is preferable to find how the partial permittivity ε_pc_ and ε_ps_ are linked to the relative partial susceptibility RX_c_ and RX_s_. Then, the model functions Φ(r_c_) and Ψ(r_sin_), constructed in [31] for RX as a combination of normal and lognormal functions in the scale 0.0 ≤ RX_c_, RX_s_ ≤ 1.0, can be used for fitting to the experimental data series “ε_cp_(r_c_)-r_c_” and “ε_ps_(r_sin_)-r_sin_”. Such an approach ensures a higher accuracy of fitting as well.

Taking into account Equations (3) and (5) provides model functions Φ1(r_c_) and Ψ1(r_sin_) for “ε_pc_(r_c_)-r_c_” and “ε_ps_(r_sin_)-r_sin_”:

Φ1(r_c_) = 1.0 + (ε_t_ − 1.0)Φ(r_c_) and

Ψ1(r_sin_) = 1.0 + (ε_t_ − 1.0)Ψ(r_sin_),

where:

(9) $$\Phi (r_{c})=\frac{1}{\sqrt{2\pi }\sigma_{1}}\int_{0}^{r_{c}}e^{-\frac{{\left(r-\mu_{1}\right)}^{2}}{2\sigma_{1}^{2}}}dr\;at\;r_{c}<r_{infl}\;and\;\;\;\Phi \left(r_{c}\right)=\frac{1}{\sqrt{2\pi }\sigma_{2}r_{c}}\int_{r_{c}}^{R_{0}}e^{-\frac{{\left[\ln\left(r\right)-\mu_{2}\right]}^{2}}{2\sigma_{2}^{2}}}dr\;at\;r_{c}\geq r_{infl},\;\Psi \left(r_{sin}\right)=1.0-\frac{1}{\sqrt{2\pi }\sigma_{1}}\int_{0}^{r_{sin}}e^{-\frac{{\left(r-\mu_{1}\right)}^{2}}{2\sigma_{1}^{2}}}dr\;at\;r_{c}<r_{infl}\;and\;\Psi (r_{sin})=1.0-\frac{1}{\sqrt{2\pi }\sigma_{2}r_{sin}}\int_{r_{sin}}^{R_{0}}e^{-\frac{{\left[ln(r)-\mu_{2}\right]}^{2}}{2\sigma_{2}^{2}}}dr\;at\;r_{c}\geq r_{infl},$$

and parameters μ_1_, μ_2_—mean values and σ_1_, σ_2_—standard deviations.

Model functions Φ(r_c_) and Ψ(r_sin_) for relationships “RX_c_(r_c_)-r_c_” and “RX_s_(r_sin_)-r_sin_” were defined in [31] as follow:

Φ(r_c_) ≈ RX_c_(r_c_) and Ψ(r_sin_) ≈ RX_s_(r_sin_). (10)

Then, the relative partial permittivity RE for cylinders and shells can be expressed from Equation (6) by Φ(r_c_) and Ψ(r_sin_):

(11) $${\mathrm{RE}}_{c}(r_{c})\;\approx \Phi 2(r_{c})\mathrm{and}{\mathrm{RE}}_{s}(r_{\sin})\;\approx \Psi 2(r_{\sin}),\;\mathrm{where}\;\Phi 2(r_{c})=\frac{1}{\varepsilon_{t}}+\frac{\chi_{t}}{\varepsilon_{t}}\Phi (r_{c})\;\mathrm{and}\;\Psi 2(r_{\sin})=\frac{1}{\varepsilon_{t}}+\frac{\chi_{t}}{\varepsilon_{t}}\Psi (r_{\sin}).$$

Model curves of the functions “RE_c_(r_c_)-r_c_” and “RE_s_(r_sin_)-r_sin_” were drawn for dielectrics of group 1 and model curves of the functions “RE_c_(r_c_)-r_c_” for dielectrics of group 2.

### 3.3. Rate of Change

At a certain radius of a cylindrical subsample r_c_, the rate of change in the quantities ε_pc_ and RE_c_ gives the increment/decrement in the quantity if r_c_ increases/decreases for such a value, which provides an increment/decrement Δk_c_ in the coverage of active area. Let us evaluate the rate of change in the partial permittivity ε_p_ and the relative partial permittivity RE at constant increments of the OSA sensor’s coverage with a subsample Δk_c_ = const. and Δk_s_ = const. The rate of change in the partial permittivity for partial cylinders and shells is calculated from Equation (3):

(12) $$\frac{∆\varepsilon_{pc}}{∆k_{c}}=\frac{∆\chi_{pc}}{∆k_{c}}\;and\frac{∆\varepsilon_{ps}}{∆k_{s}}=\frac{∆\chi_{ps}}{∆k_{s}}.$$

Taking into account Equations (5) and (10), we obtain the following:

(13) $$\frac{∆\varepsilon_{pc}}{∆k_{c}}=\chi_{t}\frac{∆{RX}_{c}(k_{c})}{∆k_{c}}\approx \left(\varepsilon_{t}-1.0\right)\frac{∆\Phi (k_{c})}{∆k_{c}}\;and\;\frac{∆\varepsilon_{ps}}{∆k_{s}}=\chi_{t}\frac{∆{RX}_{s}(k_{s})}{∆k_{s}}\approx \left(\varepsilon_{t}-1.0\right)\frac{∆\Psi (k_{s})}{∆k_{s}}.$$

The rate of change of the relative partial permittivity for cylinders and shells is calculated from Equation (11):

(14) $$\frac{∆{RE}_{c}(k_{c})}{∆k_{c}}\approx \frac{\chi_{t}}{\varepsilon_{t}}\frac{∆\Phi (k_{c})}{∆k_{c}}\;and\frac{∆{RE}_{s}(k_{s})}{∆k_{s}}\approx \frac{\chi_{t}}{\varepsilon_{t}}\frac{∆\Psi (k_{s})}{∆k_{s}}.$$

To determine the change rate in the relative partial permittivity over the radius r_c_ of active area, covered with a dielectric cylinder, the curves “ΔRE(k_c_)/Δk_c_−k_c_” were calculated at such r_c_ values, which correspond to constant increments of coverage coefficient Δk_c_ = k_c(i+1)_ − k_ci_ = const., where “k_ci_” and “k_c(i+1)_” are points on the k_c_-axis, i = 1, 2, …, I + 1, and I = k_c_/Δk_c_ = 1.0/Δk_c_. We obtain k_ci_ = (r_ci_/R_0_)^2^ from Equation (1) and the corresponding increments in the radius of a cylinder are calculated as Δr_ci_(r_c_) = $-r_{ci}\pm \sqrt{r_{ci}^{2}+R_{0}^{2}∆k_{c}}$, where i = 1, 2, …, I. Since r_c_ ≥ 0.0 mm for all 0.0 mm ≤ r_c_ ≤ R_0_ mm, the positive square-root is taken. Then, the values of increments in radius Δr_ci_ depend on r_c_ in a way, which ensures Δk_c_ = const. (similar calculations can be made for the curves “ΔRE(k_s_)/Δk_s_-k_s_” in case of shells) [31].

To illustrate the rate of change over the radius of the OSA sensor’s active area, covered partially with a dielectric cylinder, the values of $\frac{∆{RE}_{c}}{∆k_{c}}$ and $\frac{∆\varepsilon_{pc}}{∆k_{c}}$ were calculated at such values of radius r_c_, which correspond to constant increments in the coverage coefficient Δk_c_ = Δk_c5%_ = 5% [31]. Since k_c_ = S_c_/S_0_, the corresponding increment in the covered active area ΔS_c_ = Δk_c5%_S_0_ = 79.5 mm^2^ ≈ 80 mm^2^. The curves “$\frac{∆\varepsilon_{pc}}{∆k_{c}}$-r_c_” and “$\frac{∆{RE}_{c}}{∆k_{c}}$-r_c_” were drawn for dielectric materials with a significantly differing true permittivity (group 2), since, in this case, the general trends are more clearly pronounced (similar considerations are valid for the subsamples—shells).

The highest values of the rates of change max Δε_pc_/Δk_c5%_ and max ΔRE_c_/Δk_c5%_ were determined for ε_pc_ and RE_c_, and their dependence on the density and true permittivity of the PU foams, the monolithic polyurethanes, and the epoxy was investigated for cylindrical subsamples. In numerical calculations, the functions Φ(r_c_) and Ψ(r_sin_) were determined by the normalized cumulative standard functions NORMDIST(r_c_, μ_1_, σ_1_, TRUE) and LOGNORM.DIST(r_c_, μ_2_, σ_2_) of the software MS EXCEL v16.0 (Microsoft Corporation; Redmond, WA, USA). The approximating functions were determined for the dependence of the highest rate of change on the density and the true permittivity.

## 4. Results and Discussion

### 4.1. The Relative Partial Permittivity and the Relative Partial Susceptibility

**Figure 2:**
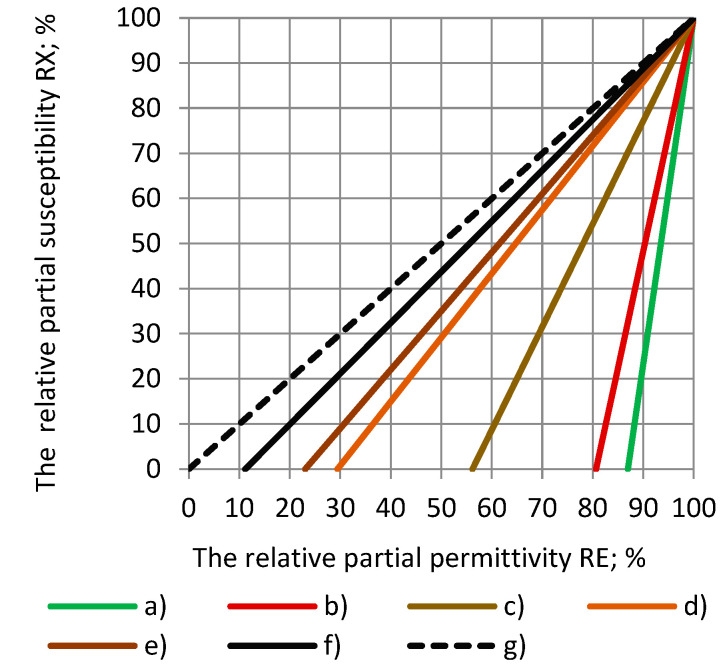
Dependence of the relative partial susceptibility RX on the relative partial permittivity RE at different values of the true permittivity: (a) SikaBlockM80, (b) SikaBlockM150, and (c) SikaBlockM450 and monolithic materials (d) Lab-made PU, and (e) PU SikaBlockM945, as well as (f) epoxy Lab975 New and (g) a hypothetical dielectric (ε_t_ = ∞).

The dependence of the relative partial susceptibility RX on the relative partial permittivity RE at different values of the true permittivity ε_t_ is given in Figure 2 for the considered dielectric materials. When ε_t_ increases, then RX → RE, and, at sufficiently high values of the true permittivity, RE ≈ RX, as seen in Equation (6). The dashed line marks the equality of RE and RX for a hypothetical dielectric with an infinitely high ε_t_: RE = RX.

It can be seen that the lower the true permittivity, the higher the difference between the corresponding RE and RX values. For the epoxy Lab975 New of a comparatively high true permittivity ε_t_ = 8.95, when RE = 90%, then RX = 89%, and it can be concluded RE ≈ RX. In such case, it is often sufficient to consider just the relative partial susceptibility.

The true permittivity of PU foams and monolithic polyurethanes is quite small, ε_t_ ≤ 4.5, in the entire range of densities, 30 kg/m^3^ ≤ ρ ≤ 1350 kg/m^3^, and the difference between RE and RX is significant; e.g., for SikaBlockM80, when RE = 90%, then RX = 25%, and, for the lab-made PU, when RE = 50%, then RX = 29%. It can be concluded RE >> RX. Therefore, in different estimations, the relative partial permittivity has to be considered besides the relative partial susceptibility in the case of the rigid PU foams.

### 4.2. The Inter-Electrode Zone

The hand-cutting technology of the subsamples yielded the uncertainty of the transversal dimensions Δr_c_ = ± 0.5 mm … ± 1.5 mm (depending on the density) due to deviations from a circular shape. The uncertainty of the concentric placement of a sample on the active area was estimated as Δ’r_c_ = ± 0.5 mm … ± 0.7 mm. A circular ring between electrodes 1 and 2, of width 2.0 mm, is referred to as the inter-electrode zone, where r_1in_ is the inner radius of electrode 1 and r_2_ is the radius of electrode 2. The inter-electrode zone comprises the gaps g1 and g2, each of width 0.55 mm, and the guard electrode of width 1.00 mm, as seen in Figure 1a, meaning that the dimensions of the gaps and the guard electrode are comparable to the uncertainties of the subsample’s transversal dimensions and placement. A measurement of the partial permittivity ε_pc_ for a subsample with a radius r_c_ provides an average of the accurate ε_pc_(r_c_) values measured at an r_c_ interval: r_c_ − (Δr_c_ + Δ^′^r_c_) ≤ r_c_ ≤ r_c_ + (Δr_c_ + Δ^′^r_c_). Therefore, the given experiment could not resolve the values of ε_pc_, which correspond to cylindrical subsamples with radii corresponding to the inter-electrode zone r_2_ < r_c_ < r_3in_, r_3in_ ≤ r_c_ ≤ r_3out_ and r_3out_ < r_c_ < r_1in_. The gaps and the guard electrode are too narrow for a more detailed characterization of the inter-electrode zone.

The partial permittivity ε_pc_ is a cumulative quantity, which increases at an increase in the radius of a cylindrical subsample. When the radius of a subsample takes values corresponding to the inter-electrode zone r_2_ < r_c_ < r_1in_, the partial permittivity has to match the following inequalities:

ε_pc_(r_2_) < ε_pc_(r_c_) < ε_pc_(r_1in_) and ε_pc_(r_c_′) > ε_pc_(r_c_) at r_c_′ > r_c_. (15)

The experiments confirmed that the experimental data points ε_pc_(r_c_) of the studied dielectric materials match the inequalities (15). Since a detailed character of the relationships “ε_pc_–r_c_” and “ε_ps_–r_sin_” in the inter-electrode zone could not be determined in the given experiments due to the above-mentioned reasons, the functions, derived from Φ(r_c_) and Ψ(r_c_), were used to model “ε_pc_–r_c_” and “ε_ps_–r_sin_” in the inter-electrode zone as well (similar considerations can be made for shells).

### 4.3. Experimental Data

**Figure 3:**
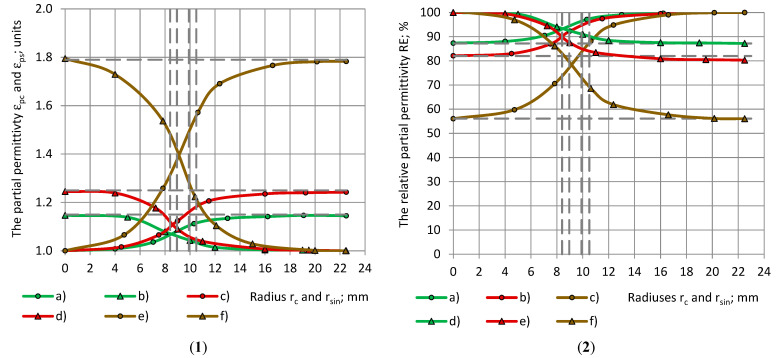
(**1**) The partial permittivity of PU foams’ cylinders and shells ε_pc_ and ε_ps_: (a), (c), (e) and (b), (d), (f) in dependence of the samples’ radiuses r_c_ (circles) and r_sin_ (triangles). (a,b) SikaBlockM80; (c,d) SikaBlockM150 and (e,f) SikaBlockM450 and (**2**) The relative partial permittivity RE(r_c_) and RE(r_sin_) of PU foams in dependence of radiuses of cylinders and shells r_c_ and r_sin_: (a,d) SikaBlockM80, (b,e) SikaBlockM150 as well as (c,f) SikaBlockM450.

Experimental data of the true (the dashed horizontals) and the partial permittivity of low- to medium-density PU foams with a similar true permittivity 1.00 < ε_t_ < 1.80 (group 1) are given in Figure 3 with a dependence on the radii of the samples and subsamples (further–samples).

**Figure 4:**
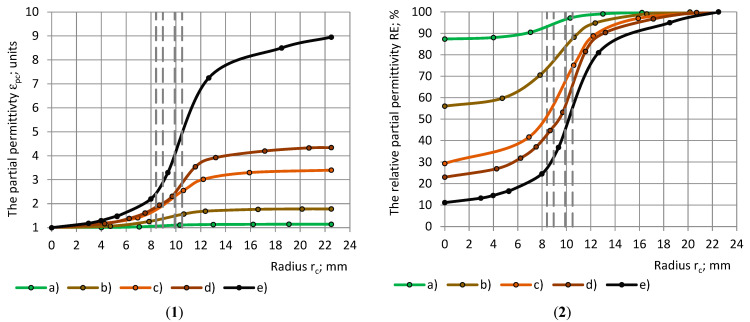
(**1**) The partial permittivity ε_pc_ in dependence of cylinder’s radius r_c_: PU foams (a) SikaBlockM80, (b) SikaBlockM450, monolithic materials (c) lab-made PU, (d) PU SikaBlockM945 and (e) epoxy Lab975 New and (**2**) The relative partial permittivity RE(r_c_) in dependence of cylinder’s radius r_c_: (a) PU foams SikaBlockM80, (b) PU foams SikaBlockM450, monolithic materials (c) lab-made PU, (d) PU SikaBlockM945 and (e) epoxy Lab975 New.

**Figure 5:**
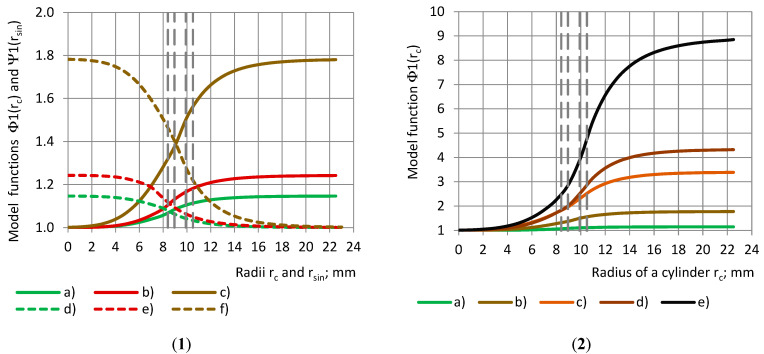
(**1**) Model functions Φ1 and Ψ1 of the partial permittivity ε_pc_ and ε_ps_ of PU foams’ cylinders and shells with a dependence on the samples’ radii r_c_ and r_sin_. Cylinders: (a), (b), and (c); shells: (d), (e), and (f). PU foams: (a) and (d) SikaBlockM80, (b) and (e) SikaBlockM150, as well as (c) and (f) SikaBlockM450 and (**2**) Model functions Φ1 of the partial permittivity ε_pc_ in dependence of cylinder’s radius r_c_: (a) PU foams SikaBlockM80, (b) PU foams SikaBlockM450, monolithic materials (c) lab-made PU, (d) PU SikaBlockM945 and (e) epoxy Lab975 New.

**Figure 6:**
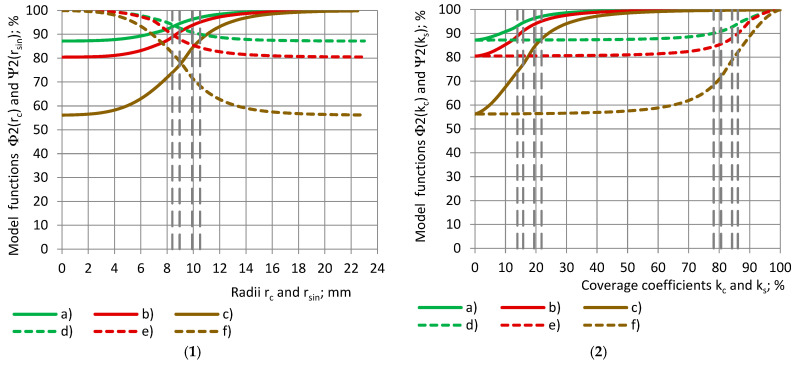
Model functions Φ2 and Ψ2 of the relative partial permittivity RE with a dependence on (**1**) samples’ radii r_c_ and r_sin_ and (**2**) coverage coefficients k_c_ and k_s_. Cylinders: (a), (b), and (c); shells: (d), (e), and (f). PU foams: (a) and (d) SikaBlockM80, and (b) and (e) SikaBlockM150, as well as (c) and (f) SikaBlockM450.

**Figure 7:**
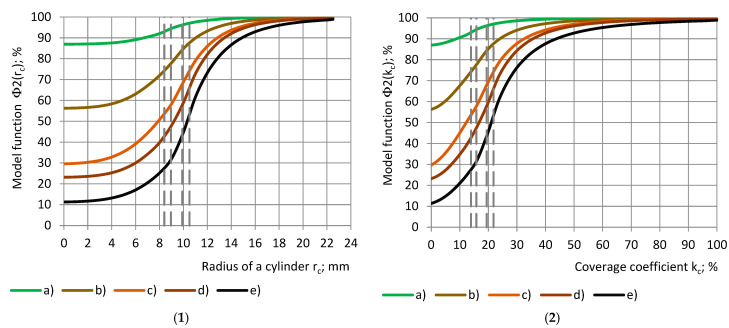
Model functions Φ2 of the relative partial permittivity RE with a dependence on (**1**) cylinder’s radius r_c_ and (**2**) coverage coefficient k_c_. (a) PU foams SikaBlockM80, and (b) PU foams SikaBlockM450, and monolithic materials (c) lab-made PU, (d) PU SikaBlockM945, and (e) epoxy Lab975 New.

**Figure 8:**
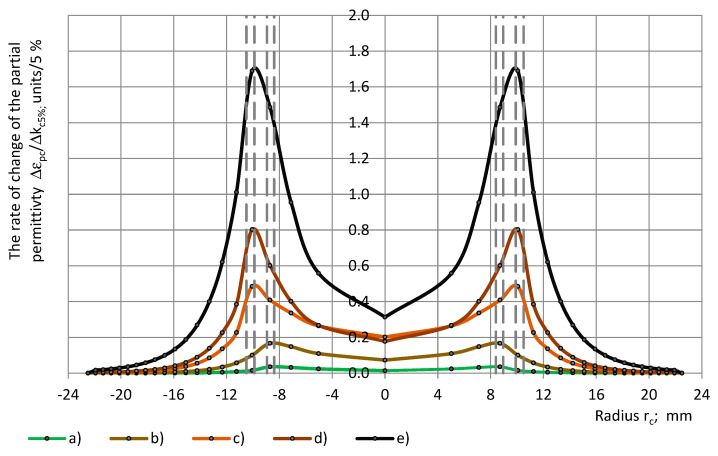
The rate of change of the partial permittivity Δε_pc_/Δk_c5%_ with a dependence on cylinder’s radius r_c_: (a) PU foams SikaBlockM80 and (b) PU foams SikaBlockM450, and monolithic materials (c) lab-made PU, and (d) PU SikaBlockM945, as well as (e) epoxy Lab975 New.

**Figure 9:**
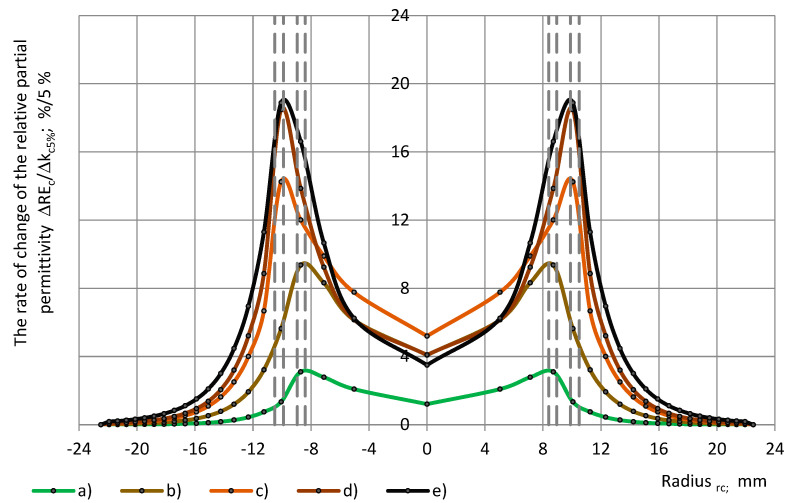
The rate of change of the relative partial permittivity ΔRE_c_/Δk_c5%_ with a dependence on cylinder’s radius r_c_: (a) PU foams SikaBlockM80 and (b) PU foams SikaBlockM450, and monolithic materials (c) lab-made PU, and (d) PU SikaBlockM945, as well as (e) epoxy Lab975 New.

The dashed verticals in Figure 3, Figure 4, Figure 5, Figure 6, Figure 7, Figure 8 and Figure 9 mark the radius r_2_ of electrode 2, the inner and outer radii r_3in_ and r_3out_ of electrode 3, and the inner radius r_1in_ of electrode 1. It can be seen that, at an increase in r_c_ from 0.0 mm to R_0_ = 22.5 mm, the partial permittivity ε_pc_ increases from 0.0 to the value of true permittivity ε_t_. The concave–convex curves “ε_pc_-r_c_” have an inflection point at r_2_ ≤ r_c_ ≤ r_1in_.

Figure 3(2) shows the relative partial permittivity RE with a dependence on the radius of the samples, cylinders, and shells, for PU foams with a similar true permittivity 1.00 < ε_t_ < 1.80. The data curves “RE(r_c_)-r_c_” follow a concave/convex pattern as well, with the inflection point in the limits r_2_ ≤ r_c_ ≤ r_1in_. When r_c_ → R_0_ = 22.5 mm (a complete sample), then ε_m_ → ε_t_. The higher the true permittivity ε_t_, the smaller the value of RE at r_c_ = 0.0 mm. For low- to medium-density PU foams (ρ = 85–415 kg/m^3^) with a similar true permittivity 1.00 < ε_t_ < 1.80, at r_c_ → 0.0 mm, the lower values of RE lie in the range 56–94%. The conclusions for the curves “RE(r_sin_)-r_sin_” for shells are similar, besides the fact that the data curves are convex/concave.

Experimental data of the partial permittivity of PU materials and epoxy Lab975 New with a significantly differing true permittivity 1.0 < ε_t_ < 9.0 (group 2) are given in Figure 4(1) with a dependence on the radius of the cylindrical samples. The character of the curves is similar to those in Figure 3.

Figure 4(2) depicts the relative partial permittivity RE with a dependence on the radius r_c_ of the cylindrical samples for PU materials and the epoxy Lab975 New. The curves “RE(r_c_)-r_c_” follow a concave/convex pattern, with the inflection point in the limits r_2_ ≤ r_c_ ≤ r_1in_. For the considered dielectric materials (having 1.0 < ε_t_ < 9.0), the lower value of RE lies in the limits 10–87%. When r_c_ → R_0_ = 22.5 mm (a complete sample), then RE → 100%.

### 4.4. Model Functions for the Dielectrics of the Groups 1 and 2

Model functions Φ1(r_c_) and Ψ1(r_sin_) of the partial permittivity for group 1 dielectrics are given in Figure 5(1) with a dependence on the radii of the samples. Figure 5(2) gives Φ1(r_c_) for the group 2 dielectrics.

Figure 6(1) shows the relative partial permittivity RE of the PU foams with a similar true permittivity with a dependence on (1) the radii r_c_ and r_sin_ of the cylinders and shells, as well as (2) the coverage coefficients k_c_ and k_s_, when the RE data are fitted by model functions Φ2(r_c_) and Ψ2(r_sin_).

It can be seen that, for each material, the curves “Φ1-r_c_” and “Ψ1-r_sin_”, as well as the curves “Φ2-r_c_” and “Ψ2-r_sin_”, are symmetrical about a straight line, which is drawn parallel to the Or_c_ (Or_sin_) axis, and which is passing through the intersection point of the curves.

Let us consider the corresponding subsamples, cylinders and shells, defined by the equality of the radii r_c_ = r_sin_ [31]. For the corresponding subsamples, when the cylindrical subsample is placed into its corresponding shell, a complete sample is obtained. It can be concluded from Figure 6(1) that, for any of the considered PU foams, the following is valid at r_c_ = r_sin_:

(16) $$\Phi 2(r_{c})+\Psi 2(r_{sin})=1.0+\frac{1.0006}{\varepsilon_{t}}.$$

For the coverage coefficients of the corresponding subsamples, k_s_ = 1.0−k_c_, and we obtain the following:

(17) $$\Phi 2\left(k_{c}\right)+\Psi 2\left(1.0-k_{c}\right)=1.0+\frac{1.0006}{\varepsilon_{t}}.$$

Figure 7 shows the model functions Φ2(r_c_) of the relative partial permittivity RE with a dependence on radius r_c_ of the cylinders and coverage coefficient k_c_. When the functions “Φ2-r_c_” are determined for the cylinders, the curves for the shells “Φ2-r_sin_” can be calculated from Equation (17):

(18) $$\Psi 2(r_{sin})=\Phi 2(r_{c})-\left(1.0+\frac{1.0006}{\varepsilon_{t}}\right).$$

All conclusions, made for the experimental data curves of ε_t_ and RE, remain valid for the model functions Φ1, Ψ1 and Φ2, Ψ2.

### 4.5. The Rate of Change

The rate of change of the partial permittivity Δε_pc_/Δk_c_ with a dependence on the radius of a cylinder r_c_ is depicted in Figure 8 for PU materials and the epoxy (group 2).

Since the partial permittivity ε_pc_ is a dimensionless quantity, its rate of change Δε_pc_/Δk_c5%_ is calculated over the radius r_c,_ in units, per constant increments of the coverage coefficient Δk_c_ = 5%. It means, when at a certain radius r_ci_, the coverage coefficient increases for Δk_c_ = 5%, the corresponding increment in the radius is Δr_ci_(r_ci_) = $-r_{ci}\pm \sqrt{r_{ci}^{2}+R_{0}^{2}∆k_{c}}$, where i = 1, 2, …, I + 1 and I = k_c_/Δk_c_ = 1.0/Δk_c_ [31], and the partial permittivity increases for Δε_pc_ units. The increments Δr_c_ (corresponding to the markers on the curves in Figure 8 and Figure 9) depend on r_c_, in a way, which ensures constant increments in the coverage coefficient Δk_c_ = Δk_c5%_ = 5%. It can be concluded that the rate of change in units per 5% increment in the coverage coefficient Δk_c5%_ is the highest for dielectrics (the PU materials and the epoxy) with the highest true permittivity.

It can be seen that the rate of change for the PU materials is the highest at the inter-electrode zone: 0.03 units/5% ≤ max Δε_pc_/Δk_c5%_ ≤ 0.81 units/5%. The scatter in the location of max Δε_pc_/Δk_c5%_ can be explained by a comparatively high uncertainty of the samples’ transversal dimensions (Section 4.2) and low values of the partial permittivity of the PU foams. In the centre of the active area, the rate of change is 2–4 times lower. As the radius r_c_ increases above approx. 14 mm, the Δε_pc_/Δk_c_ decreases rapidly.

Figure 9 gives the rate of change in the relative partial permittivity ΔRE_c_/Δk_c_ over the radius of the sensor’s circular concentric zone, which is covered with a dielectric cylinder of radius r_c_.

While Figure 3 and Figure 4 depict the integral relationships “ε_pc_(r_c_)-r_c_” and “RE_c_(r_c_)-r_c_”, Figure 8 and Figure 9 show the differential character of the ε_pc_ and RE_c_ change at a partial coverage of the active area. The rate of change in units per 5% increment in the coverage coefficient Δk_c5%_ is the highest for PU materials with the highest true permittivity.

Conclusions about the rate of change in the relative partial permittivity ΔRE_c_/Δk_c5%_ are similar to those about Δε_pc_/Δk_c5%_. ΔRE_c_/Δk_c5%_ is the highest at the inter-electrode zone 2.6%/5% ≤ max ΔRE_c_/Δk_c5%_ ≤ 18.4%/5% and max ΔRE_c_/Δk_c_ = 19% for the epoxy Lab975 New. In the centre of the active area, the rate of change is 2.5–5 times lower. As the radius r_c_ increases above approx. 14 mm, the ΔRE_c_/Δk_c_ decreases.

To obtain proper data for the rate of change in the centre of the active area, a sufficient amount of experimental data is needed for cylinders of a small radius r_c_ and shells with a small inner radius r_sin_. The partial permittivity of these subsamples is highly sensitive to deviations in the transversal cross-sectional shape from circularity and a concentric location of the samples on the active area. High-precision turning and milling technologies should be tested to make accurate circular cylinders with a small diameter, and precise drilling to make accurate shells with a small inner radius.

The partial permittivity curves (Figure 3 and Figure 4) as well as the curves of the derived quantities (Figure 8 and Figure 9) depend both on the parameters of the dielectric material and the active area of the OSA sensor. It can be seen that the overall character of the rate of change in the partial permittivity with a dependence on the subsample’s radius (equal to the radius of the covered area) is comparable to that of the surface charge density distribution curves for a circular two-electrode OSA sensor, calculated from the Laplace equation [14]. The effective empirical manifestation of the active area of the given OSA sensor is similar to that of a two-electrode one. At the same time, theoretical considerations suggest a more complex character (several maxima) of the functions “ε_pc_-r_c_” and “ε_pc_-r_sin_” at the inter-electrode zone [5,19,20]. Knowledge of the functions “ε_pc_-r_c_” and “ε_pc_-r_sin_” might facilitate the determination of the transcapacitance of the OSA sensor with three electrodes on the active surface.

More research is necessary on alternative experimental methods for the estimation of the functions “ε_pc_-r_c_” and “ε_pc_-r_sin_” in the inter-electrode zone. Increasing the accuracy of the subsample’s circular shape is not a solution, since at least 3–5 data points are needed for each gap and the guard electrode to estimate the character of curves. That determines the increment in a subsample’s radius: (a) Δr_c_ = 1.0 mm/5 = 0.2 mm in the region of the guard electrode and (b) Δr_c_ = 0.5 mm/5 = 0.1 mm in the region of gaps. The reliability of such experimental data is questionable, because increments 0.1 mm and 0.2 mm are comparable to the dimensions of the characteristic elements of the PU foams’ structure and to the uncertainty of the concentric placement of a subsample on the active area Δ′r_c_ = ± 0.5 mm … ± 0.7 mm. Sensitivity of the spectrometer can be insufficient as well.

An indirect approach can be tested: making several sensors, each having three electrodes and two gaps on the active surface, all of a width exceeding the uncertainties of subsamples’ transversal dimensions by at least several times. Based on the partial permittivity data at the guard electrode and gaps, becoming narrower from one sensor to the other, the character of the curves could be estimated for small dimensions. At the same time, there are many practical situations, when knowledge of the effective empiric manifestation of the narrow inter-electrode zone is sufficient.

### 4.6. Dependence of on PU Foams’ Properties

**Figure 10:**
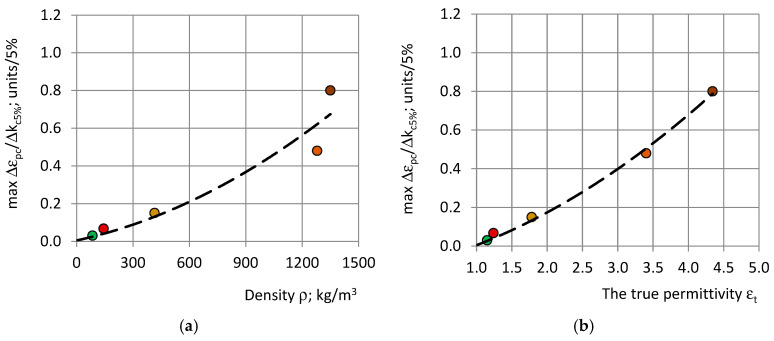
The highest rate of change max Δε_pc_/Δk_c5%_ with a dependence on (**a**) density ρ and (**b**) the true permittivity ε_t_ for the following: PU foams: SikaBlockM80 (green), SikaBlockM150 (red), and SikaBlockM450 (khaki), and monolithic polyurethanes: lab-made PU (orange) and PU SikaBlockM945 (brown). The dashed lines mark the trendlines.

The highest rate of change max Δε_pc_/Δk_c5%_ and max ΔRE_c_/Δk_c5%_ characterizes the slopes of the curves “ε_pc_-r_c_” and “RE_c_-r_c_” at the inflection point, at a 5% increment in k_c_, as seen in Figure 3. The values of max Δε_pc_/Δk_c5%_ with a dependence on the density and the true permittivity of the PU materials are shown in Figure 10.

max Δε_pc_/Δk_c5%_ increases according to nonlinear trends, approximated with second-order polynomials:

max Δε_pc_/Δk_c5%_ ≈ 0.0000002 ρ^2^ + 0.0002178 ρ + 0.0047443 and R^2^ ≈ 0.93; max Δε_pc_/Δk_c5%_ ≈ 0.0278 ε_t_^2^ + 0.0861 ε_t_ − 0.1092 and R^2^ ≈ 0.99; (19)

where max Δε_pc_/Δk_c5%_ is calculated in units per 5% and R^2^ is the coefficient of correlation. The lowest value of max Δε_pc_/Δk_c5%_ corresponds to PU foams SikaBlockM80: max Δε_pc_/Δk_c5%_ ≈ 0.03 and the highest to monolithic PU: max Δε_pc_/Δk_c5%_ ≈ 0.8.

**Figure 11:**
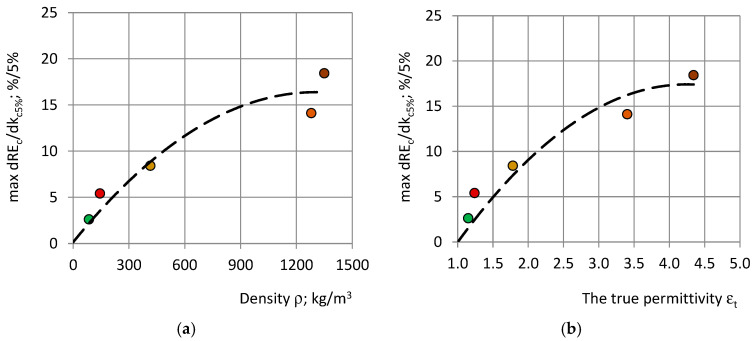
The highest rate of change max ΔRE_c_/Δk_c5%_ with a dependence on (**a**) density ρ and (**b**) the true permittivity ε_t_ for the following: PU foams: SikaBlockM80 (green), SikaBlockM150 (red), and SikaBlockM450 (khaki), and monolithic polyurethanes: lab-made PU (orange) and PU SikaBlockM945 (brown). The dashed lines mark the trendlines.

The values of max ΔRE_c_/Δk_c5%_ with a dependence on the density ρ and the true permittivity ε_t_ of the PU materials are shown in Figure 11.

With an increase of density and the true permittivity max ΔRE_c_/Δk_c5%_ increases according to nonlinear trends:

max ΔRE_c_/Δk_c5%_ ≈ −0.0000096 ρ^2^ + 0.0249191 ρ + 0.1446782 and R^2^ ≈ 0.94; max ΔRE_c_/Δk_c5%_ ≈ −1.6558 ε_t_^2^ + 14.0606 ε_t_ − 12.4399 and R^2^ ≈ 0.90; (20)

where max ΔRE_c_/Δk_c5%_ is calculated in % per 5%. For the lab-made monolithic polyurethane and the industrially produced PU SikaBlockM945, the max ΔRE_c_/Δk_c5%_ reaches the highest value ≈ 14–18%, while, for the light-weight PU foams SikaBlockM80, it is only around 3%.

## 5. Conclusions

A novel experimental study was carried out dealing with the partial permittivity of rigid PU foams and monolithic polyurethanes by means of a capacitive circular OSA sensor, in a wide range of foams’ densities. An original and effective method was elaborated to determine the model functions of the obtained experimental data. The numerical estimation of the rate of change in the partial permittivity revealed that the highest rate of change corresponds to the inter-electrode zone. The study showed the appropriateness of rigid PU foams for the investigation of the partial permittivity due to the monotonously increasing permittivity at an increasing density. It permitted us to identify that the highest rate of change depends on the density and the true permittivity of rigid PU foams in a nonlinear mode, approximated with second-order polynomials.

The overall character of the rate of change in the partial permittivity with a dependence on the radius of the covered area was found to be comparable to that of the surface charge density distribution curve, estimated for a circular two-electrode OSA sensor theoretically. It suggests that the rate of change is proportional to the average surface charge density over increments in the covered area. The experimental results on the partial permittivity can be useful in the performance evaluation and design of the optimal proportions of the capacitive circular OSA sensors, as well as in the verification of the corresponding mathematical models. The basic concepts of the study remain valid for capacitive rectangular OSA sensors, which widens the fields of application.

The developed methodology is envisaged for dielectrics and is inappropriate for the conductive PU foams’ composites, used in ELMG shielding. Further studies are necessary on the partial coverage at the other absolute and relative dimensions of the electrodes and gaps of the OSA sensors. More dielectric materials have to be tested to validate the generality of the proposed model functions.

## Figures and Tables

**Table 1:** Characteristics of the dielectrics; 1 kHz [21,22].

N	Dielectric Material	Density ρ; kg/m^3^	The True Permittivity ε_t_	N	Dielectric Material	Density ρ; kg/m^3^	The True Permittivity ε_t_
1	SikaBlockM80	85	1.15	4	Lab-made PU	1280	3.40
2	SikaBlockM150	144	1.24	5	PU SikaM945	1350	4.34
3	SikaBlockM450	415	1.78	6	Epoxy LAB975 New	708	8.95

## Data Availability

The data are contained within this article.
